# Synergizing Process to Optimize Myocardial Infarction Patient Outcomes: Results of the Myocardial Infarction Delhi Primary Angioplasty Study (MIDAS)

**DOI:** 10.1016/j.jscai.2025.103667

**Published:** 2025-10-14

**Authors:** Roopa Salwan, Alexandra Lansky, Kalpana Singh, Kritya Dubey, Surbhit Sharma, Sameer Mehta

**Affiliations:** a Interventional Cardiology, Max Super Speciality Hospital, New Delhi, India; b Section of Cardiovascular Medicine, Department of Internal Medicine, Yale School of Medicine, New Haven, Connecticut; c Hamad Medical Corporation, Doha, Qatar; d Emergency Department, Max Super Speciality Hospital, New Delhi, India; e Max Super Speciality Hospital, New Delhi, India; f Lumen Foundation & GLOW, Lumen Global, Miami, Florida

**Keywords:** door-to-balloon time, ischemic heart disease, primary percutaneous coronary intervention, reperfusion therapy, ST-segment elevation myocardial infarction

## Abstract

**Background:**

The prevalence and case fatality of ischemic heart disease in India has increased over the past 2 decades. ST-segment elevation myocardial infarction (STEMI) accounts for up to 60% of all myocardial infarctions in India, with only 25% to 40% of patients undergoing primary percutaneous coronary intervention (PPCI). A national access gap persists in India that needs to be bridged with the implementation of in-hospital processes and regional systems of collaborative care.

**Methods:**

The single-center, observational, retrospective Myocardial Infarction Delhi Primary Angioplasty Study (MIDAS) was conducted at Max Super Specialty Hospital (New Delhi, India) between January 1, 2015, and December 31, 2019. All patients presenting to the emergency department with ongoing chest discomfort suggestive of ischemia and ST elevation on electrocardiogram were included. PPCI, initiated by a “CODE STEMI” team activation call, was employed as a single reperfusion strategy to treat all patients and minimize system delays. Systematic data collection of door-to-balloon (D2B) time regular audit, feedback, and multidisciplinary team engagement was implemented to refine processes and improve efficiency. The consistency of the process in maintaining D2B time during day and night hours, as well as on weekends and holidays was evaluated. Data from the electronic health record were collected and summarized to evaluate the impact of processes on in-hospital patient outcomes.

**Results:**

A total of 887 patients meeting eligibility criteria were included in the analysis, of whom 45.2% were admitted during off hours, weekends, or holidays, and 92.3% underwent PPCI, 2.8% underwent PPCI followed by CABG, 1.8% underwent CABG, and 2.6% required medical therapy. The median D2B was 51 minutes (interquartile range, 40-60 minutes), and on holidays the mean D2B was 55 minutes (interquartile range, 40-75 minutes). The overall mortality was 5.2%.

**Conclusions:**

This study demonstrates that implementing a team-based standardized protocol can achieve guideline-recommended D2B times in a diverse STEMI population presenting to a percutaneous coronary intervention–capable hospital. Protocols reduce variation in care and enable the delivery of equitable high-quality care consistently across diverse demographic groups.

## Introduction

Cardiovascular disease (CVD) is the leading cause of mortality and morbidity in India, with a prevalence of 14% in urban areas, 7.4% in rural areas,^1^ and 53.4% of crude deaths due to CVD are among people younger than 70 years.^2^ Ischemic heart disease (IHD), is the most prevalent form of CVD in India, with estimated 23.8 million cases. Lifestyle changes with urbanization, aging, and population growth have led to a rapid epidemiological transition in the entire country wherein noncommunicable diseases exceed communicable diseases. The high mortality is a result of increased exposure to risk factors at a younger age, aging of the population, inadequate preventive care, and lack of access to effective and equitable care.^3^ Estimates from the Global Burden of Disease 2019 study suggest that by 2050 in South Asia, IHD will account for a majority of age-standardized cardiovascular mortality burden (141 deaths per 100,000 population).^4^

Myocardial infarction (MI) is a severe, often the first, and potentially fatal clinical manifestation of IHD that requires rapid recognition and early treatment. In India, ST-segment elevation myocardial infarction (STEMI) accounts for 37% to 60% of all MI,^5^^,^^6^ with an estimated more than a million cases a year.^7^ STEMI is a time-sensitive condition in which appropriate and rapid use of reperfusion therapy is crucial to preserve myocardial function and improve patient outcomes. Reperfusion can be achieved pharmacologically by fibrinolytic therapy or mechanically by primary percutaneous coronary intervention (PPCI). Patients treated by fibrinolysis can subsequently be transferred safely to a percutaneous coronary intervention (PCI)-capable hospital for further treatment (pharmacoinvasive approach).^8^ PPCI is the most effective method to provide early, complete, sustained reperfusion and preserve myocardial function. Patients have a shorter hospital stay, fewer readmissions, and lower incidence of recurrent ischemia, intracerebral bleeding, and death as compared to those treated by fibrinolytic therapy.^9^^,^^10^ All leading cardiology societies have recommended PPCI as a class Ia intervention in STEMI, provided experienced operators can perform the procedure within 90 minutes of reporting to a PCI-capable center.^7^^,^^11^^,^^12^ The advantage of PPCI over fibrinolysis is lost if the in-hospital delay is more than 120 minutes.^13^, ^14^, ^15^ A 1-hour delay in door-to-balloon (D2B) is associated with a 55% increase in 1-year mortality, whereas, an hour delay in symptom onset to door time is associated with a 4% increase in 1-year mortality.^16^ Guidelines also recommend (level Ia) that all communities should create and maintain a regional care network for STEMI patients that includes patient education to enable early symptom recognition, in-hospital care process, and prehospital care through close collaboration between PCI-capable and non–PCI-capable hospitals and ambulance services. Existing resources should be used efficiently to enhance processes to provide timely, coordinated, and consistent care.^11^^,^^12^

In the past 2 decades, the health care landscape in India has evolved with improved infrastructure in public and private hospitals. In metropolitan cities, there has been an increase in the number of PCI-capable hospitals, with 70% of tertiary care services being private. Despite reasonable access to cath lab-equipped hospitals,^17^ PPCI is performed in only 25% to 40% of patients.^18^^,^^19^ The majority of patients living in cities self-present to the hospital. Delays and deficiencies in care are associated with high mortality and morbidity, especially in the younger population.

Interventions that focus on utilizing existing resources, enhancing hospital processes, and developing functional teams are more likely to accelerate the delivery of care, improve patient outcomes, and reduce mortality. Subsequently, collaboration with regional hospitals to integrate capabilities would enable to develop STEMI system of care.^20^^,^^21^

Strategies that focus on processes within the hospital have been shown to significantly reduce D2B time in performance of PPCI for patients with STEMI.^22^ We adopted and implemented these by developing STEMI care protocols for 24 × 7 × 365 PPCI and operationalizing CODE STEMI—a single call to activate the team. The retrospective Myocardial Infarction Delhi Primary Angioplasty Study (MIDAS) provides real-world insights into care processes and clinical outcomes in a diverse STEMI population in a rapidly developing country, India. The study evaluates the consistency of hospital processes at all times, during day and night hours, on weekends and holidays, and we sought to assess the impact of D2B time on patient outcome and in-hospital mortality, as well as equity of care related to elderly, women and economically weaker sections (EWS, patients without insurance cover) is also studied.

## Methods

### Study design

A single-center, observational, retrospective study of all patients presenting to the emergency department with chest discomfort suggestive of ischemia with ST elevation on electrocardiogram (ECG) between January 1, 2015, and December 31, 2019. The study was conducted at Max Super Speciality Hospital ( New Delhi, India), a private tertiary care 525 bed, teaching hospital with 3 cardiac cath labs, staffed by a team of 7 consultant interventional cardiologists, 6 attending interventional cardiologists, and an integrated emergency, cardiothoracic surgical services and critical care unit supported by nephrology, neurology, and gastroenterology units. A clinical pathway with a clear goal of treating all eligible STEMI patients with a single reperfusion strategy of PPCI within 90 minutes of arrival at the hospital was developed and operationalized. All cardiology consultants agreed to the strategy and approved the protocol. CODE STEMI enabled cath lab activation by a single call from emergency to a central page operator who paged the interventional cardiologist, cath lab staff to expedite the procedure, and duty manager to provide logistic support in the admission process. There was a STEMI program lead clinician in emergency and cardiology to give feedback, train and develop teams, and a lead administrator responsible for arranging admission and handling financial clearance. By hospital policy, 10% of in-hospital patients belonging to EWS were treated without payment. Data were systematically collected, the variance was tracked, and rectified, and with continuous feedback, the system constantly evolved over time.

Clinical presentation, demographics, procedural, and outcomes were collected from the electronic medical record. The in-hospital processes measures, clinical, and procedural details were systematically collected and summarized. The study was conducted in accordance with New Drugs and Clinical Trials Rules, 2019, chapter IV, and National Ethical Guidelines for Biomedical and Health Research Involving Human Participants, ICMR 2017. Study participants were identified by a specific code number in their subsequent records. Thus, the confidentiality of the information that concerns the participants was completely secure and strictly confidential. This protocol was reviewed by the institutional ethics committee and approved. The institutional ethics committee gave a consent waiver as this was a retrospective study.

### Study population

Patients aged >18 years with ongoing chest pain or discomfort or ischemia equivalent symptom lasting more than 20 minutes to 24 hours, with ECG showing ST elevation (STE) of >1 mm in the contiguous chest or limb leads or in V2, V3 >1.5 mm in women, >2 mm in men >40 years, >2.5 mm in men <40 years and/or left bundle branch block, STE aVr and/or V1 with ST depression >6 leads, ST depression V1 to V3 and STE V7 to V9, STE V3R V4 R, presenting to hospital emergency, for whom CODE STEMI was called were included in the study. Patients with pain lasting over 12 hours with dynamic ECG changes, suggesting ongoing ischemia, hemodynamic instability, or arrhythmia were also included.

### Treatment protocol

**Figure 1:**
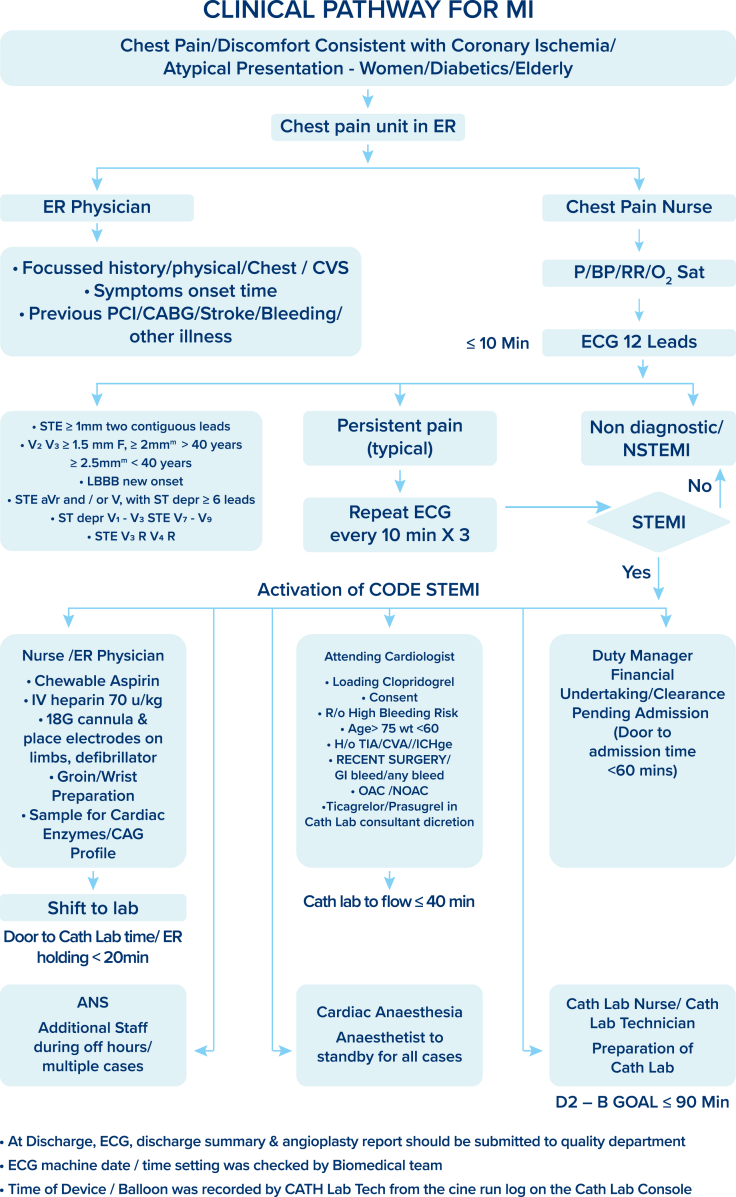
**Clinical pathway for myocardial infarction (MI): Standardized protocol with defined role and responsibility of each team member with a clear goal to achieve door-to-balloon time of <90 minutes.** ANS, additional nursing superintendent; BP, blood pressure; CABG, coronary artery bypass graft; CAG, coronary angiography; cath lab, cardiac catheterization lab; CVA, cerebrovascular accident; CVS, cardiovascular system; ECG, electrocardiogram; ER, emergency room; GI, gastrointestinal; IC Hge, intracranial hemorrhage; LBBB, left bundle branch block; NOAC, non-vitamin K antagonist oral anticoagulant; NSTEMI, non–ST-segment elevation myocardial infarction; OAC, oral anticoagulation; PCI, percutaneous coronary intervention; RR, respiratory rate; STE, ST elevation; STEMI, ST-segment elevation myocardial infarction; TIA, transient ischemic attack.

A single reperfusion strategy of PPCI was employed to treat all patients with STEMI. A single call, CODE STEMI, was operationalized to activate the entire team. A standardized protocol was developed with a defined role for all team members and was implemented to provide 24 × 7 × 365 PPCI and achieve D2B in <90 minutes. Figure 1 summarizes the clinical pathway for MI.

An interdisciplinary team comprising emergency physicians, cardiologists, emergency room (ER) and catheterization laboratory nursing staff and technicians, cardiovascular surgeons, cardiac anesthetists, quality managers, and administrators provided a shared goal of timely care to patients with STEMI. There was a designated chest pain area in the ER and a dedicated STEMI nurse who ensured an ECG was recorded within 10 minutes of the arrival of a patient with chest pain. The ER physician took a focused history, did the primary assessment, and interpreted the ECG. In the event of STEMI, the ER physician was empowered to call CODE STEMI wherein the call center informed all members of the team. A green corridor was created to eliminate any delay in transferring the patient from the ER to the Cath lab after registration, without the need for financial clearance. The cath lab was staffed at all hours with on-site attending consultant interventional cardiologists and other appropriate staff; the interventional consultant stayed on-site or arrived within 30 minutes of CODE STEMI. Regular boot camps, mock drills, and training were conducted to maintain the skill and coordination of the team to provide seamless care.

Time to treatment was measured as D2B time. This interval was defined as the time from the first hospital arrival to the time of first balloon inflation or use of another device such as a thrombus aspiration (TA) catheter or stent, inclusive of the emergency department arrival to cath lab and cath lab to balloon or device time.

A daily report of STEMI cases, door-to-ECG, emergency to cath lab time, D2B time, and discharge treatment was collected by the STEMI secretary. Monthly meetings of the lead emergency consultant, quality manager, cardiologist, and administrator were held to audit processes, verify and validate the implementation of protocols, identify deficiencies, and take feedback to improve the process.

The CODE STEMI patient lists with door-to-cath lab, cath-to-balloon and D2B time were maintained prospectively for patients by the quality team and STEMI secretary. The time of admission was divided into regular working hours (day: 8:00 AM to 7:59 PM), off-hours (night: 8:00 PM to 7:59 AM), holidays, and weekends. From hospital records, time of admission (day or night), duration of stay, and mode of payment (self-pay, third-party administrator, EWS, corporate insurance) were recorded. The shortest driving distance from the patient’s home to the hospital was calculated using Google Maps as a proxy measure for proximity to PPCI care access.

The collection and compilation of clinical data for the study were performed by extracting data from the electronic health records by dedicated personnel and validated by a single reviewer. Data collected included pain onset to door time, comorbidity (hypertension, diabetes mellitus, family history of premature coronary artery disease [CAD], smoking, previous MI, PCI, coronary artery bypass surgery [CABG], extent of coronary artery disease [1,2,3 vessel obstructive CAD], or nonobstructive CAD, procedure performed coronary angiography or PPCI, CABG, intra-aortic balloon pump [IABP], temporary pacemaker implant, ventilator support, use of TA catheter, requirement of dialysis), echocardiographic defined ejection fraction (<35%, 36%-45%, >45%), in-hospital mortality, and discharge medications.

### Statistical analysis

Descriptive statistics were used to summarize the demographic and clinical characteristics of the study participants. Continuous variables are presented using mean, SD, median, and IQR. Categorical variables are summarized as frequencies and proportions. χ^2^ tests were used for statistical association between categorical variables. Continuous variables are summarized in boxplots for door-to-cath, cath-to-balloon, and D2B, illustrating the median, quartiles, and potential outliers. All analyses were performed using Stata 17 statistical software (StataCorp LLC) with a significance level set at a *P* value <.05 (2-tailed test).

## Results

During the study period, CODE STEMI was activated for 900 patients. Eight patients left against medical advice, there were 2 false calls (non–ST-segment elevation myocardial infarction), and 3 patients had left bundle branch block with normal coronaries; these were excluded from the analysis. There were 4 patients who had cardiac arrest before angiography could be done. A total of 883 patients were transferred to the cath lab for coronary angiography; 20.1% of patients were women. Of these patients, 819 (92.3%) underwent PPCI, 25 (2.8%) patients underwent PPCI followed by CABG in the same admission, 16 (1.8%) patients were treated with CABG after initial stabilization, and 23 (2.6%) patients required medical therapy (Central Illustration).

**Central Illustration:**
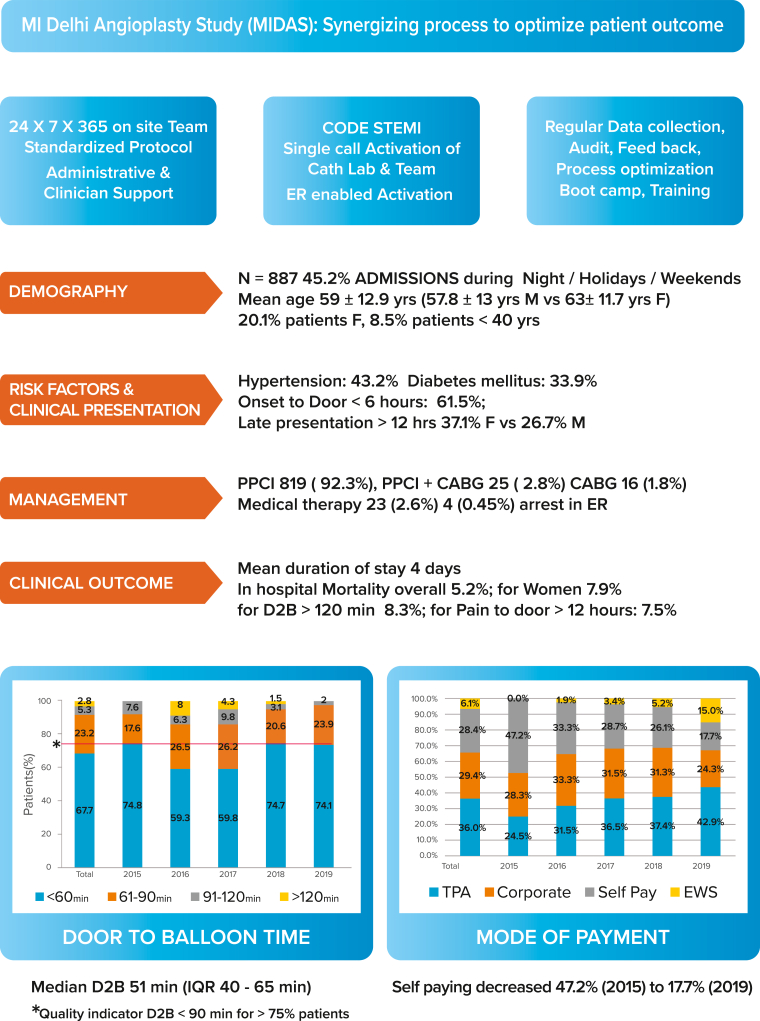
**Myocardial Infarction Delhi Angioplasty Study (MIDAS): summary of the study design, patient characteristics, treatment given, in-hospital patient outcomes, process consistency in achieving door-to-balloon (D2B) <90 minutes in >75% of patients, and change in the mode of payment from 2015 to 2019.** CABG, coronary artery bypass graft; ER, emergency room; EWS, economically weaker section; PPCI, primary percutaneous coronary intervention; STEMI, ST-segment elevation myocardial infarction; TPA, third-party administrator.

The average age of patients was 59 ± 12.9 years (57.8 ± 13.0 years for men and 63 ± 11.7 years for women, *P* < .001); 26.4% of patients were younger than 50 years, and 8.4% were younger than 40 years. Hypertension (43.2%) and diabetes mellitus (33.9%) were the most prevalent risk factors. The prevalence of smoking was low at 6.9% (8.5% in men vs 1.6% in women; *P* = .001). Multiple risk factors were more prevalent in women (32.6%) and patients undergoing CABG (39%). The median duration of chest discomfort before reporting to emergency was 4 hours. A majority of patients (61.5%) had reported to emergency within 6 hours of onset, 24.8% having come within 2 hours of onset of chest discomfort. Women were more likely to report more than 12 hours after onset of pain than men (37.1% vs 26.7%; *P* = .004). The demographic profile and associated risk factors in patients are outlined in Table 1.

**Table 1:** Demographic and risk factor profile of patients.

Characteristics	Total (N = 887)	Women (n = 178)	Men (n = 709)	*P* value
Age, y	59.0 ±12.9	63.7 ±11.7	57.8 ±13.0	<.001
Age groups, y				<.001
<30	12 (1.4)	–	12 (1.7)	
31-40	63 (7.1)	2 (1.1)	61 (8.6)	
41-50	159 (17.9)	27 (15.2)	132 (18.6)	
51-60	251 (28.3)	42 (23.6)	209 (29.5)	
61-70	235 (26.5)	57 (32.0)	178 (25.1)	
71-80	118 (13.3)	32 (18.0)	86 (12.1)	
>80	49 (5.5)	18 (10.1)	31 (4.4)	
Risk factors				
Hypertension	383 (43.2)	97 (53.4)	288 (40.6)	.002
Diabetes mellitus	301 (33.9)	83 (46.6)	218 (30.7)	<.001
Family history of premature CAD	17 (1.9%)	2 (1.1%)	15 (2.1%)	.39
Smoking	61 (6.9)	2 (1.1)	59 (8.3)	.001
Multiple risk factors	217 (24.5)	58 (32.6)	159 (22.4)	.005
Prior MI	21 (2.4)	5 (2.8)	16 (2.3)	.66
Prior PTCA	19 (2.1)	5 (2.8)	14 (2.0)	.49
Prior CABG	4 (0.5)		4 (0.6)	.32
Pain-to-door time, h				.004
<2	220 (24.8)	30 (16.9)	190 (26.8)	
2-6	326 (36.8)	60 (33.7)	266 (37.5)	
6-12	86 (9.7)	22 (12.4)	64 (9.0)	
>12	255 (28.7)	66 (37.1)	189 (26.7)	
Distance from hospital, km				.52
<5	360 (40.6)	71 (39.9)	289 (40.8)	
5-10	262 (29.5)	48 (27.0)	214 (30.2)	
>10	265 (29.9)	59 (33.1)	206 (29.1)	

Data shown as n (%) or mean ± SD.

CABG, coronary artery bypass grafting; CAD, coronary artery disease; MI, myocardial infarction; PTCA, percutaneous transluminal coronary angioplasty.

### Hospital process measures: Time to treatment

**Figure 2:**
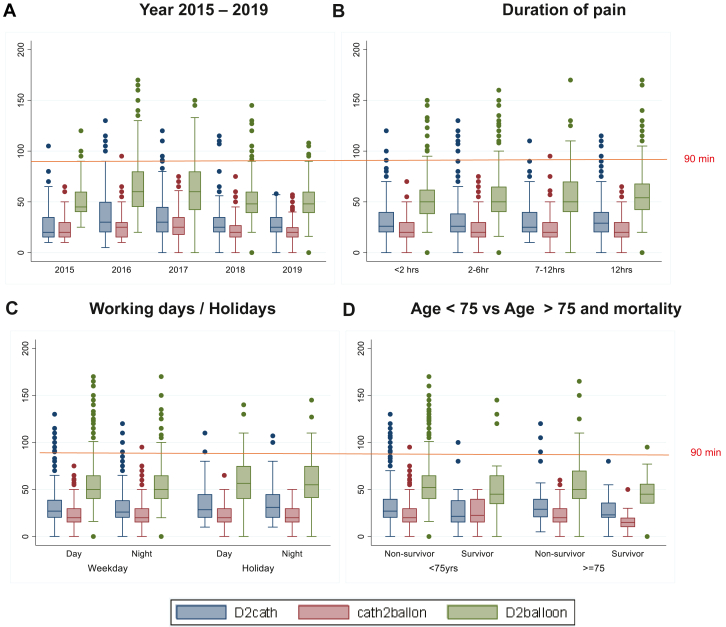
**Box plot for process measure (time to treatment door-to-balloon [D2B]).** By (**A**) year of procedure 2015 to 2019, (**B**) duration of pain, (**C**) working day (day/night) and holidays (day/night), and (**D**) elderly >75 years (survivor and nonsurvivor) vs <75 years (survivor and nonsurvivor). The horizontal line in each box indicates the median and the top and bottom indicate the interquartile range; the horizontal line at 90 minutes is the guideline-recommended time to treatment. Guideline-recommended D2B less than 90 minutes was consistently achieved each year, during working hours (day/night) (D2B; *P* = .20) and holidays (day/night) (D2B; *P* = .20, irrespective of duration of pain <2 hours, 2 to 6 hours, 6 to 12 hours, >12 hours (D2B; *P* = .40) and for elderly patients aged >75 years survivors and nonsurvivors as compared to patients aged <75 years (survivors and nonsurvivors) (D2B; *P* = 0.41).

Table 2 provides a detailed summary of in-hospital processes, including time trends analyzed by year, time of day, sex, and patient characteristics. There were 484 (54.8%) admissions during working hours, 279 (31.6%) during night hours, and 120 (13.6%) on holidays and weekends. The median door-to-cath time was 27 (20-40) minutes, the median cath-to-balloon time was 20 (15-30) minutes, and the median D2B time was 51 (40-65) minutes. The D2B time <90 minutes was achieved in 92% of patients during daytime, 91.7% of patients during night hours, and 87.1% of patients on holidays. The emergency hold time was marginally higher on holidays (30 minutes vs 20 minutes). D2B time <90 minutes in >75% of patients was consistently achieved for women, elderly patients, patients of EWS, and patients who presented late (12 to 24 hours) with ongoing discomfort. Figure 2 depicts the time intervals for cardiac intervention procedures by year, time of day, duration of pain, and for the elderly (age >75 years).

**Table 2:** In-hospital processes by year, time of day, and patient characteristics.

Category	Door-to-cath, min (n = 883)		Cath-to-balloon, min (n = 844)		Door-to-balloon, min (n = 844)	
	Median (IQR)	Mean ± SD	Median (IQR)	Mean ± SD	Median (IQR)	Mean ± SD
Total	27.0 (20.0, 40.0)	32.2 ± 19.3	20.0 (15.0, 30.0)	23.3 ± 13.0	51.0 (40.0, 65.0)	55.3 ± 25.7
Year						
2015	20.0 (15.0, 35.0)	28.0 ± 16.7	20.0 (15.0, 30.0)	25.2 ± 10.9	45.0 (40.0, 62.0)	53.7 ± 21.2
2016	30.0 (20.0, 50.0)	39.4 ± 25.4	25.0 (15.0, 30.0)	26.1 ± 12.9	60.0 (45.0, 80.0)	65.4 ± 30.5
2017	30.0 (20.0, 45.0)	36.9 ± 23.2	25.0 (17.5, 35.0)	26.5 ± 14.6	60.0 (42.0, 80.0)	60.4 ± 30.5
2018	25.0 (20.0, 35.0)	29.5 ± 15.7	20.0 (15.0, 27.0)	20.7 ± 12.2	48.0 (39.0, 60.0)	50.1 ± 22.2
2019	25.0 (20.0, 35.0)	28.4 ± 11.6	20.0 (15.0, 25.0)	20.4 ± 12.6	48.0 (39.0, 60.0)	49.3 ± 19.1
Day						
Day (n = 484)	27.0 (20.0, 40.0)	32.3 ± 19.2	20.0 (15.0, 30.0)	24.5 ± 11.6	52.0 (40.0, 65.0)	57.0 ± 23.7
Night (n = 279)	26.0 (20.0, 39.0)	31.5 ± 18.1	20.0 (15.0, 30.0)	25.5 ± 13.6	52.0 (41.0, 65.0)	56.8 ± 23.3
Holidays (n = 120)	30.0 (20, 45)	34.9 ± 21.3	20.0 (15.0, 30.0)	24.7 ± 11.6	55.0 (40.0, 75)	60.7 ± 25.2
Sex						
Men	26.0 (20.0, 38.5)	31.4 ± 18.3	20.0 (15.0, 30.0)	23.4 ± 12.8	50.0 (40.0, 65.0)	54.6 ± 24.8
Women	30.0 (20.0, 45.0)	35.6 ± 22.4	20.0 (15.0, 30.0)	22.9 ± 14.0	53.0 (40.0, 70.0)	58.2 ± 29.0
Patient characteristics						
EWS	27.5 (20.0, 36.0)	29.6 ± 10.6	20.0 (15.0, 30.0)	22.4 ± 12.2	52.0 (44.0, 60.0)	55.2 ± 18.5
Elderly	28.0 (20.0, 40.0)	32.6 ± 20.3	20.0 (15.0, 30.0)	22.0 ± 12.9	50.0 (40.0, 70.0)	54.7 ± 27.1
Pain >12 h	29.0 (20.0, 40.0)	33.5 ± 20.3	20.0 (15.0, 30.0)	23.9 ± 12.8	55.0 (42.0, 68.0)	57.2 ± 25.9

EWS, economically weaker section.

### Angiographic and procedural details

On angiography, single-vessel disease was present in 41% of patients, double-vessel disease in 27.8%, triple-vessel disease in 26.96%, and 2.6% of patients had nonobstructive disease. Ejection fraction was <30% in 11.3%, 31% to 45% in 53.6%, and >45% in 34.8% of patients. The use of IABP was 11.3% in 2015 which declined to 5.7% over the study period. TA catheter use declined from 65.1% in 2015 to 19.3% in 2019 (overall use 30.4%). Ventilator support was required in 13.6% of patients. Dialysis was required in 0.6% of patients. Table 3 highlights the outcomes of patients, presenting a comprehensive analysis of their clinical results and associated factors.

**Table 3:** Patient outcomes.

Characteristics	Total (N = 883)	2015 (n = 106)	2016 (n = 162)	2017 (n = 178)	2018 (n = 211)	2019 (n = 226)	*P* value
Single-vessel disease	370 (41.0)	57 (53.8)	79 (48.8)	64 (36.0)	83 (37.6)	87 (36.8)	.003
Double-vessel disease	253 (27.8)	21 (19.8)	45 (27.8)	58 (32.6)	61 (27.7)	68 (28.1)	.25
Triple-vessel disease	241 (26.6)	26 (24.5)	35 (21.6)	54 (30.3)	64 (29.1)	62 (25.9)	.36
Nonobstructive CAD	19 (2.1)	2 (1.9)	3 (1.9)	2 (1.1)	3 (1.4)	9 (3.9)	–
Ejection fraction							.017
<30%	100 (11.3)	14 (13.2)	19 (11.7)	19 (10.7)	22 (10.3)	26 (11.4)	
31%-45%	476 (53.9)	48 (45.3)	108 (66.7)	98 (55.1)	107 (51.2)	115 (50.4)	
>45%	307 (34.8)	44 (41.5)	35 (21.6)	61 (34.3)	82 (38.5)	85 (38.1)	
Thrombus aspiration	266 (30.0)	69 (65.1)	72 (44.4)	53 (29.8)	28 (13.1)	44 (19.3)	<.001
IABP	46 (5.2)	12 (11.3)	6 (3.7)	4 (2.2)	11 (5.2)	13 (5.7)	<.001
TPI	27 (3.0)	9 (8.5)	4 (2.5)	5 (2.8)	4 (1.9)	5 (2.2)	.016
Ventilator	121 (13.6)	12 (11.3)	19 (11.7)	26 (14.6)	31 (14.6)	33 (14.5)	.83
Dialysis	5 (0.6)	1 (0.9)	–	1 (0.6)	1 (0.5)	2 (0.9)	.80
Death	46 (5.2)	7 (6.6)	8 (4.9)	7 (3.9)	11 (5.2)	13 (5.7)	.89
Duration of stay, d	4.0 ± 6.6	3.8 ± 3.1	3.7 ± 3.4	4.8 ± 10.8	4.1 ± 7.6	3.7 ± 3.5	.46

Values are n (%) or mean ± SD.

CAD, coronary artery disease; IABP, intra-aortic balloon pump; TPI, temporary pacemaker implant.

### In-hospital mortality, discharge treatment, and reimbursement

The overall mortality was 5.2% in the hospital. The mortality of patients aged less than 50 years was 3.4%. The mortality rate was higher for women (7.9%), patients undergoing CABG (9.8%), those who presented >12 hours of pain (7.5%), those with D2B >120 minutes (8.3%), and for elderly (>75 years; 16.98%). The mean hospital stay was 4 days. All patients undergoing interventional procedures received dual antiplatelet therapy at discharge. The majority of patients undergoing PPCI were treated with more potent P2Y12 blockers (ticagrelor 66.7%, prasugrel 12.14%). At discharge, the prescription of statin was 82.2%, beta-blockers 76.3%, and ACEi or ARB 40.3%.

There were 54 (6.1%) patients from the EWS who were treated without any payment to the hospital, 28.4% of patients paid out of pocket, 29.4% had corporate insurance, and 36% third third-party insurance. Over 5 years’ time, the proportion of self-paying patients decreased from 47.2% to 17.7% patients (*P* = .001), reflecting an increase in the uptake of individual insurance (Central Illustration). A majority of patients (69.8%) lived within 10 km of the hospital.

## Discussion

This retrospective study evaluated the impact of a standardized clinical care pathway on timely triage and round-the-clock (24 × 7 × 365) PPCI, aiming to achieve short D2B times in a diverse STEMI patient population. PPCI was performed in 92.8% of patients, 4.6% underwent CABG, and 2.6% received medical therapy alone. Patients were treated consistently with high-quality care in accordance with guidelines.^23^ This was achieved by strategic planning, streamlined processes, unified reperfusion pathways, optimal resource utilization, interdisciplinary collaboration, and scientific rigor in implementation. Clinical surveillance, feedback, boot camps, and regular training helped maintain team cohesion even amid high attrition and workforce shifts in private hospitals.

The standardized protocol was effectively implemented across all shifts, with 45.2% of patients presenting on weekends, holidays, or night hours. Although holiday emergency hold times were longer (30 minutes vs 20 minutes), over 75% of patients achieved D2B times under 90 minutes. It was achieved for a diverse vulnerable population, including elderly patients, women, those presenting late (ongoing pain >12 hours after onset of pain), and those from EWS. This finding is significant in demonstrating that standardized protocols reduce variation and enable the delivery of equitable care uniformly across diverse demographic groups.

Historically, thrombolysis has been the main reperfusion pathway in studies published from India. The CREATE registry^5^ reported thrombolysis in 58.5% and PCI in only 8% of patients. In the Kerala ACS registry,^6^ 41.4% of patients were treated by thrombolysis and 12.9% by PCI. Data from the EPICOR Asia^18^ identified a large implementation gap between guideline recommendations and actual care, despite having the most PPCI-capable centers. India had the longest delay (20.9 hours) and the lowest rate of PPCI (24.8%). The TN STEMI study,^19^ that implemented a protocol-driven hub and spoke model, reported an overall reperfusion rate of 90.1% in STEMI patients, with an increased reperfusion rate by PPCI from 21.8% to 40.7% and by pharmacoinvasive approach from 13.1% to 20.1%. D2B time reported in the TN STEMI study was 100 minutes^19^ and in the Kerala PPCI registry 1.2 hours.^24^ In the HP ACS registry, 2.2% of patients were treated by PCI in 2013, that increased to 21.9% in 2018.^25^^,^^26^ In the recently published NORIN STEMI registry from New Delhi, 72% of patients underwent PCI within 71 hours after onset of pain, with a door-to procedure time of 8 hours. This study was an observational study and did not report any in-hospital process optimization protocol.^27^

A quality STEMI procedure starts with an efficient STEMI process. Expanding health care infrastructure can translate to improved patient outcomes with the implementation of in-hospital processes and high-quality care by adequately trained teams. Most errors result from ineffective systems, not individual failures. Data from the Global Burden of Disease have shown CVD deaths in low-middle-income countries , particularly in South Asia, account for 33% of amenable deaths in the total health system, of which 84% were caused by use of poor quality health services. The total low-middle-income countries poor quality mortality was 82 deaths per 100,000 population.^28^

Trials from European countries with organized ambulance systems and short transfer times showed PPCI reduced mortality as compared to fibrinolytic therapy regardless of presentation delay; however, in-hospital delay in PPCI was associated with higher mortality.^29^^,^^30^ At that time, health care in the US was fragmented with intense competition between individuals and hospital systems. Centers of excellence with high volume PPCI, committed, dedicated teams developed and extended collaboration to non–PCI centers to develop regional STEMI care networks based on the hub and spoke model and implementation of standardized protocols.^31^, ^32^, ^33^ Another approach, shown to be feasible and effective, was EMS-driven triage to STEMI receiving centers, bypassing ER when they were full and overcrowded.^34^ The rapid transport of STEMI patients has been shown to be safe and effective, and collaborative care facilitated treatment of “eligible but untreated” patients by improving access to PPCI. Each STEMI regional system of care has been developed according to the unique geographic, political, and socioeconomic conditions of their areas.

The largest STEMI registry of southeast Asia, KAMIR,^35^ was developed in 2005 and was based on the collaborative effort of STEMI centers treating patients with PPCI. In China, the STEMI care project planned improvement over 10 years. The initial focus was on in-hospital process optimization followed by regional, city-wide, and national collaboration to create STEMI networks.^36^

The demographics of our study identify a young population with a mean age of 59 years, with women being 5 years older than men and a higher prevalence of hypertension (53.4% vs 40.6% in men) and diabetes (46.6% vs 30.7% in men). These findings are similar to the Kerala ACS registry.^6^ The prevalence of smoking in our group was lower than that reported in other Indian studies (6.9% vs 22.5-69%).^5^^,^^6^^,^^18^^,^^19^^,^^24^, ^25^, ^26^, ^27^^,^^37^^,^^38^ It is likely that smoking was underreported in patient records and could not be verified, as ours was a retrospective study.

The study’s interventional practices aligned with updated guidelines.^39^^,^^40^ There was a significant decrease in the use of TA catheters (65.1% in 2015 to 19.3% in 2019) and IABP use (from 11.3% to 5.7%). Most of the patients were treated with more potent and rapid P2Y12 receptor blockers (ticagrelor in 66.7% and prasugrel in 12.1%). The mortality in patients treated within 90 minutes was 4.6% compared to 8.3% for D2B over 120 minutes and 7.5% for those with ongoing chest discomfort >12 hours. This was comparable to mortality reported in the Kerala PPCI registry (4.2%)^24^ and compares favorably with other Indian studies; CREATE (8.6%), KERALA ACS (8%), NORIN STEMI (6%) and HP India ACS (11%), NE Registry (11.76%),^37^ and AIIMS (12.9%).^38^

Financial constraints have been a limiting factor in providing optimal care in low-middle-income countries. During the course of our study, we found an increase in the uptake of health insurance and a reduction in the self-paying population. Constraints of medical debt are real; however, they should not withhold delivery of life-saving treatment. In metropolitan cities with a high density of cath labs, the process and outcome-driven care must be broadly implemented, which will likely require central governmental policy and monitoring to enable the delivery of patient-centered, outcome-driven high-quality care.

### Limitations

Our study is from a single center providing care to a limited population. Data collection was retrospective and the clinical and procedural details were limited to what could be retrieved from the electronic health record.

## Conclusion

This study demonstrates the feasibility and clinical impact of implementing a protocol-based 24 × 7 × 365 PPCI program to treat patients with STEMI in New Delhi, India. Public health policy designed to align and integrate STEMI care on a national level can improve patient outcomes by providing established timely life-saving treatment.
